# Role of suberoylanilide hydroxamic acid and dapagliflozin on Cx43 gene expression in diabetic cardiomyopathy rat’s model

**DOI:** 10.1038/s41598-026-49323-3

**Published:** 2026-05-19

**Authors:** Mariam R. Abubeah, Heba M. Iraqy, Dalia A. Elgamal, Enas Ahmed Hamed

**Affiliations:** 1 Department of Medical Physiology, Assiut University, Assiut, Egypt; 2 Department of Basic Medical Sciences, Badr University in Assiut, Assiut, Egypt

**Keywords:** Cardioprotective, Connexin-43, Dapagliflozin, Diabetic cardiomyopathy, Epigenetics, Suberoylanilide hydroxamic acid, Biochemistry, Cardiology, Cell biology, Diseases, Endocrinology, Molecular biology

## Abstract

Diabetic cardiomyopathy (DCM) is a major cause of death in diabetic patients. Recent studies suggest that diabetic cardiomyopathy suppresses electrical cell-to-cell coupling mediated by connexin-43 (Cx43) channels deterioration. However, there remains a clear gap in understating of pathophysiology of diabetic cardiomyopathy. This study aims to understand DCM’s mechanisms, and the protective role of histone deacetylase inhibitors (Suberoylanilide hydroxamic acid, SAHA) and sodium glucose cotransporter 2 inhibitors (Dapagliflozin) in DCM development. An experimental study conducted on type 2 diabetes mellitus (T2DM) rats (induced by high fat diet and once Streptozotocin injection; 35 mg/kg), involving 40 rats divided into four groups, control, T2DM, T2DM+SAHA (SAHA: 5 mg/kg/day), and T2DM+Dapa (Dapa:1 mg/kg/day). Experiment duration was 8 weeks. Blood samples were drawn for measurement of cardiac enzymes. Heart was dissected and used for measuring oxidative stress markers; Cx43 expression; and histological analysis. The results show that SAHA and Dapa significantly decreased malondialdehyde, creatine kinase-MB and lactate dehydrogenase levels, while significantly elevating levels of superoxide dismutase compared with T2DM group. Cardiac Cx43 mRNA and protein expression were significantly higher in T2DM+Dapa group versus T2DM group; and higher in T2DM+SAHA group versus all other groups. Histopathological lesion scoring significantly decreased in T2DM+SAHA and T2DM+Dapa versus T2DM; and in T2DM+SAHA versus T2DM+Dapa. In conclusion, this study revealed potential benefits of SAHA and Dapa in protecting against DCM. SAHA demonstrated comparatively greater effects than dapagliflozin in preserving gap junction integrity and attenuating diabetic cardiomyopathy development, potentially through both antioxidant and epigenetics mechanisms, while Dapa enhances glycemic control although these findings are limited to a single-dose experimental model.

## Introduction

Type 2 diabetes mellitus (T2DM) is a chronic, non-communicable, multisystem disease characterized by hyperglycemia and insulin resistance (IR), often accompanied by dyslipidemia. T2DM is associated with increased mortality, largely due to cardiovascular complications, particularly diabetic cardiomyopathy (DCM)^1^. The pathogenesis of DCM involves multiple interrelated mechanisms, including oxidative stress (OS), inflammation, accumulation of advanced glycation end products, and epigenetic modifications^2^. These processes contribute to structural and functional cardiac abnormalities, including impaired electrical signaling^3^.

Gap junctions are specialized intercellular channels that facilitate direct electrical and metabolic communication between cardiomyocytes. These structures are primarily composed of connexins, with connexin-43 (Cx43) being the most abundant isoform in ventricular myocardium^3^. In healthy hearts, Cx43 is localized at intercalated discs (IDs), ensuring coordinated electrical conduction. However, in DCM, alterations in Cx43 expression and distribution—such as reduced expression and lateralization—have been linked to impaired electrical coupling and arrhythmogenesis^3^.

Histone deacetylases (HDACs) are key regulators of gene expression through epigenetic modification of chromatin structure. Increased HDAC activity has been implicated in the progression of metabolic and cardiovascular diseases, including diabetes mellitus^4^. Suberoylanilide hydroxamic acid (SAHA), a pan-HDAC inhibitor, has demonstrated anti-inflammatory, antifibrotic, and antioxidative effects in various experimental models^4^. Additionally, HDAC inhibition has been reported to enhance gap junction communication and increase Cx43 expression in certain cellular systems^5^.

Sodium-glucose cotransporter-2 inhibitors (SGLT2is), such as dapagliflozin (Dapa), are widely used antidiabetic agents with well-documented cardioprotective effects^6^. Beyond glycemic control, SGLT2is improve cardiac remodeling, reduce oxidative stress, and attenuate inflammation^6^. Emerging evidence suggests that these agents may also influence Cx43 expression and localization in cardiac tissue, thereby contributing to improved electrical coupling^7^.

Despite growing evidence linking Cx43 remodeling to DCM, the mechanisms regulating its transcription and localization in the diabetic heart remain incompletely understood. Furthermore, the relative contributions of epigenetic modulation versus metabolic-targeted therapies in restoring gap junction integrity have not been directly compared. To date, no studies have evaluated the comparative effects of HDAC inhibition and SGLT2 inhibition on Cx43 expression in DCM.

This study aimed to elucidate the impacts of T2DM on the gene expression of cardiac Cx43. Highlight the possible protective effect of in vivo administration of SAHA and Dapa for 8 weeks on cardiac Cx43 gene expression, morphometric analysis of cardiac injury and remodeling, and on levels of oxidative stress markers in the cardiac homogenate as well as glycemic control, lipid profile, and cardiac enzymes in serum of T2DM rat model.

## Materials and methods

### Animals

Forty adult male Albino rats aged 8 weeks weighing 250–300 gm were recruited from the Animal House of the Faculty of Medicine, Assiut University, Egypt. They were kept under suitable laboratory conditions of temperature (25 ± 1 °C), light (12 h light/dark cycle), and humidity (60%) with *ad libitum* access to distilled water and standard rat diet for one week before the experiment for acclimatization.

### Chemicals

Streptozotocin (STZ) (Catalog# S0130) was purchased from Sigma‑Aldrich (St. Louis, MO, USA). Dapa (AstraZeneca, Cairo, Egypt) was obtained from a local pharmacy in Assiut, Egypt. SAHA (Catalog# S1047) was bought from Selleck Chemicals, USA.

### Ethical approval

Rats were manipulated in accordance with the Animal Research Reporting of In Vivo Experiments (ARRIVE) guidelines. The research was approved by the Ethical Committee of the Faculty of Medicine, Assiut University, Assiut, Egypt (approval# 04–2024-200835; 11/6/2024).

### Sample-size, randomization and blinding

The sample size was determined a priori using G*Power software (version 3.1.9.7). A power (1 − β) of 0.95 and an alpha (α) error probability of 0.05 were applied. The analysis was performed using the F-tests family, with the statistical test specified as a one-way ANOVA: fixed effects, omnibus. An anticipated effect size (f = 0.7) was estimated based on previous studies assessing myocardial Cx-43 expression, OS markers and histological remodeling in STZ-induced T2DM models. For a one-way ANOVA comparing four experimental groups, the analysis indicated that a total sample size of 40 animals (*n* = 10 per group) would be sufficient to detect statistically significant differences. This sample size also allows for potential attrition associated with long-term high-fat diet feeding and STZ induction protocols. Animals were randomly assigned to experimental groups using a simple randomization technique (computer-generated sequence). To minimize bias, the investigators performing the histological, biochemical, and molecular assays were blinded to the treatment assignments until the final data analysis was completed.

### Induction of T2DM

After one week of laboratory acclimatization, the rats were randomly divided into 4 groups (*n* = 10 each). The three T2DM groups (30 rats) were fed a high fat diet (HFD) for two weeks prior to a single injection with low dose of STZ (35 mg/kg/i.p.). The HFD was prepared by adding ground beef tallow to rat pellets and the final composition of HFD for the T2DM groups was as follows: 40% fat, 38% carbohydrate, 18% protein, and 2% fiber^8^. Confirmation of T2DM induction was carried out 72 h following STZ injection by measuring blood glucose levels using a glucometer. Rats with fasting blood glucose levels ≥ 250 mg/dl were considered diabetic and included in the study^8^. At the end of the experimental period (11 weeks in total), animals were anesthetized with isoflurane in accordance with institutional ethical guidelines and sacrificed by cervical dislocation. The HFD/STZ model reliably produced characteristic T2DM phenotypes comparable to humans, in addition to inducing DCM as previously described^9^. HFD for two weeks followed by a single low dose of STZ was sufficient to induce DCM in rats within a period of 3–12 weeks^10^.

## Experimental design

Animals were randomly allocated to experimental groups using a simple randomization method. The rats were equally divided into 4 groups (*n* = 10 each): negative control group: Rats in this group had daily access to standard laboratory chow (75% carbohydrates, 18% protein, 4% fiber and 2% fat) and water *ad libitum* for the duration of the experiment. After 3 weeks, the control group was injected i.p. with the same volume of STZ vehicle (0.1 M citrate buffer). For the next eight weeks, the control group received daily i.p. injections containing equal amounts of solvent used as the vehicle for the SAHA group (10% dimethyl sulfoxide, 45% polyethylene glycol-400, 45% saline). T2DM group: T2DM was induced as previously described, and rats continued on HFD for another 8 weeks^9^. T2DM+SAHA group: T2DM rats were injected daily with SAHA (5 mg/kg/i.p.) dissolved in (10% dimethyl sulfoxide, 45% polyethylene glycol-400, 45% saline) for 8 weeks while they remained on HFD^11^. T2DM+Dapa group: T2DM rats received daily dapagliflozin (1 mg/kg/day orally) dissolved in distilled water for 8 weeks while they remained on HFD^12^. The body weights of the rats were recorded at the beginning of the experiment and weekly throughout the experiment duration. Body weight gain percentage was calculated as final body weight minus initial body weight, divided by the initial body weight and multiplied by 100.

### Dissection of the heart

After sacrifice, the heart was quickly dissected, washed and its weight was recorded. Cardiac index was calculated by dividing heart weight by final body weight and multiplied by 100. Ventricles were sectioned from the heart and split into three parts. Part of the ventricle was homogenized in phosphate-buffered saline to prepare 10% (w/v) homogenate using a homogenizer (Omni International, Kennesaw, GA, USA) and used for measurement of OS biomarkers. A part of the left ventricle was washed with ice-cold saline; snap-frozen in liquid nitrogen and stored at − 80 °C for real-time polymerase chain reaction (RT-qPCR) assays. The final part of the left ventricle was fixed in 10% formalin for histological examination^13^. Histopathological and morphometric analyses were performed in a double-blinded manner, where the investigators were unaware of group allocation during assessment.

### Biochemical analysis

At the end of the experiment, blood samples were obtained from retro-orbital veins into a plain tube. Blood was centrifuged at 3000 rpm at 4 °C for 5 min to get the serum which was then stored at − 20 °C in aliquots until use. Serum insulin was determined using ELISA method (Catalog# KE20008, Proteintech Group, Illinois, USA). Assessment of IR was calculated by using the homeostatic model assessment of IR (HOMA-IR) index, a validated method for assessing insulin sensitivity, according to the following formula: HOMA-IR = Fasting glucose (mg/dl) X fasting insulin (µU/L)/405^14^. Lipid profiles were measured by an enzymatic method using kits obtained from BioSystems (Barcelona, Spain) as total cholesterol (TC) (Catalog# 11828), triglyceride (TGs) (Catalog# 11805), low-density lipoprotein cholesterol (LDL-C) (Catalog# 11585) and high-density lipoprotein cholesterol (HDL-C) (Catalog# 11557). Atherogenic index was calculated according to the following formula: Atherogenic index = Log (TGs/HDL-C)^14^. Detection of OS markers was done using colorimetric kits obtained from Bio-diagnostic (Egypt) as malondialdehyde (MDA) (Catalog# MD 25 29) and superoxide dismutase (SOD) (Catalog# SD 25 21). While, creatine kinase-MB (CK-MB) (Catalog# CSB -E14403r) and lactate dehydrogenase (LDH) (Catalog# 1001260) were determined using kits purchased from CUSABIO Technology LLC, Houston, USA; and SPINREACT, Barcelona, Spain; respectively.

### Real-Time polymerase chain reaction (RT-qPCR) analysis of Cx43

Ribonucleic acid extraction (RNA) extraction was performed in an RNase-free environment using the RNeasy Mini Kit (Catalog# 74104, Qiagen, Germany) in Animal Reproduction Research Institute, Giza, Egypt. Then RNA was reverse-transcribed using a High-Capacity cDNA Reverse Transcription Kit with RNase Inhibitor (Catalog# 4374966, Thermo Fisher scientific, USA). RT-qPCR reaction was performed using Maxima SYBR Green qPCR Master Mix (Catalog# K0251, Thermo-Fisher Scientific, USA). The following primer sets were used for Cx43 (Forward–5′-CTCACGTCCCACGGAGAAAA‐3′, Reverse–5′‐CGCGATCCTTAACGCCTTTG‐3′); and glyceraldehyde 3-phosphate dehydrogenase (GAPDH) was used as an internal control (Forward–5′‐GGGTGTGAACCACGAGAAAT‐3′, Reverse–5′‐ ACTGTGGTCATGAGCCCTTC‐3′)^15^. The relative expression levels of Cx43 versus GAPDH were calculated using the 2^−ΔΔCt^ method, a standard approach for quantitative gene expression analysis and were represented as the fold change relative to the control group; its value was 1^15^.

### Histological studies

Formalin-fixed cardiac specimens were fixed in paraffin, sectioned at (4 μm) by a microtome. Then slides were stained with hematoxylin and eosin (H&E) for morphological examination, Masson’s Trichrome for evaluation of fibrosis, and immunohistochemical staining^16^.

### Immunohistochemical studies

After rehydration, slides were incubated with 3% hydrogen peroxide to inhibit endogenous peroxidase activity. For antigen retrieval, slides were immersed in 10mM sodium citrate buffer. Afterwards sections were incubated with the primary antibody Cx43 (Cell Signaling Technology, USA, #3512S, 1:100) overnight at 4°C^17^. The universal staining kit Protaqs^®^ suprema PolycColor3 with horseradish peroxidase (Quartett, Berlin, Germany) was used following the manufacturer’s instructions. After completion of the reactions, sections were then counter stained using Mayer’s hematoxylin. For positive control, lung tissues were used^18^. For negative control, slides were processed following the previous steps, but the primary antibody was excluded, no immunoreactivity was present in these sections^18^.

### Morphometric analysis

A digital camera attached to a Leica universal microscope was used. And computerized image analysis software Fiji (ImageJ, version 1.2; WS Rasband, National Institute of Health, Bethesda, MD) was utilized to obtain the following measurements: cardiomyocyte cell diameters^19^, percentage area of collagen fibrosis, and percentage area of positive immunoreactivity for Cx43. For semi-quantitative analysis: previously described scoring systems^13,20,21^ were modified to create a single system that accurately represents the current pathological changes occurring in DCM. These changes are described as follows: Degeneration was defined as disorganization of myocardial fibers with loss of fiber continuity. Fibrosis was evaluated as increased collagen deposition. Vacuolation was defined as empty areas either perinuclear (halos) or scattered within the cytoplasm. Degeneration, fibrosis and vacuolation were each given a score according to the percentage of affected area per visual field: Grade 0 = no alterations were present, Grade 1 = involvement of 1–15% of myocardium, Grade 2 = involvement of 16–25%, Grade 3 = involvement of 26–50%, Grade 4 = involvement of 51–75%, and Grade 5 = involvement of > 76%. Inflammation was defined as infiltration by lymphocytes or polymorphonuclear leukocytes. The number of lymphocytes and polymorphonuclear leukocytes were counted per visual field and scored as follows: Grade 0 = 1–10 cells/field, Grade 1 = 11 to 20 cells/field, Grade 2 = 21 to 40 cells/field, Grade 3 = 41 to 60 cells/field, Grade 4 = > 61 cells/field. Vascular congestion was evaluated as excessive accumulation of blood within a vessel with or without dilatation. Grade 0 = no congestion was present, Grade 1 = presence of vascular congestion. Hypertrophy was defined as cell diameters exceeding 20 μm. Grade 0: cell diameter = ≤ 20 μm, Grade 1: cell diameter = 21–22 μm, Grade 2: cell diameter = 23–24 μm, Grade 3: cell diameter = 25–26 μm, Grade 4: cell diameter = > 27 μm. Cardiac tissues were evaluated in a double-blind manner. And each heart was inspected for these pathological changes in 10 visual non-overlapping fields. For each individual heart, a mean score for each variable was determined. These scores were then added together to obtain a single value per heart. Finally, the mean values of the group were calculated.

### Statistical analysis

Values were analyzed utilizing SPSS version 22 (Statistical Package for Social Sciences, IBM Corp., Armonk, New York, USA). The normality of data distribution was checked by Shapiro-Wilk Test. The data was expressed as mean ± standard deviation for normally distributed parametric data and median (25–75 percentile) for abnormally distributed parametric values. Data were analyzed using the appropriate tests, OneWay ANOVA for comparison of normal distribution parametric data, and Kruskal-Wallis H test for abnormal distribution parametric data. Post hoc multiple comparisons were adjusted using Tukey’s test for parametric data and Mann–Whitney U test for non-parametric data. A (*p*) value of less than 0.05 was considered to represent a statistically significant difference.

## Results

### Biological indices

The final body weights were significantly decreased in all three T2DM groups compared with the control group. The percentage change in body weight gain was significantly decreased in the T2DM group compared with the control group and significantly elevated in the T2DM+Dapa compared with the T2DM group. Meanwhile, heart index (%) was significantly increased in all three T2DM groups (Table 1).

**Table 1:** Biological indices in different studied groups.

Groups Parameters	Control	T2DM	T2DM+SAHA	T2DM+Dapa
Initial body weight	275.70 ± 50.19	234.50 ± 35.62	247.17 ± 32.82	225.71 ± 29.62
Significance	-	^*a*^*p*=0.214	^*a*^*p*=0.519, ^*b*^*p*=0.945	^*a*^*p*=0.077, ^*b*^*p*=0.978, ^*c*^*p*=0.769
Final body weight	404.50 ± 50.31	255.67 ± 48.03	294.83 ± 57.60	310.86 ± 78.85
Significance	-	^*a*^*p*<0.0001	^*a*^*p*=0.007, ^*b*^*p*=0.668	^*a*^*p*=0.018, ^*b*^*p*=0.360, ^*c*^*p*=0.962
Weight gain (grams)	128.80 ± 61.47	21.17 ± 42.41	47.67 ± 65.64	83.86 ± 55.86
Significance	-	^*a*^*p*=0.007	^a^*p*=0.053, ^b^*p*=0.856	^a^*p*=0.410, ^b^*p*=0.233, ^c^*p*=0.677
*Weight gain (%)	46.52 (32.06–70.91)	6.07 (−5.27-27.35)	8.71 (0.79–42.54)	41.46 (16.44–54.73)
Significance	-	^*a*^*p*=0.007	^*a*^*p*=0.083 ^*b*^*p*=0.522	^*a*^*p*=0.283 ^*b*^*p*=0.046 ^*c*^*p*=0.153
*Heart weight (grams)	1.30 (1.30–1.50)	1.20 (1.15–1.60)	1.50 (1.25–1.65)	1.30 (1.00 −1.50)
Significance	-	^*a*^*p*=0.254	^*a*^*p*=0.222 ^*b*^*p*=0.250	^*a*^*p*=0.534 ^*b*^*p*=1.000 ^*c*^*p*=0.281
*Heart index (%)	0.34 (0.30–0.37)	0.47 (0.45–0.48)	0.50 (0.36–0.61)	0.45 (0.34–0.54)
Significance	-	^*a*^*p*=0.002	^*a*^*p*=0.023 ^*b*^*p*=0.684	^*a*^*p*=0.019 ^*b*^*p*=0.426 ^*c*^*p*=0.567

Normally distributed data expressed as mean ± standard deviation and significance between groups was calculated using One-Way ANOVA followed by Tukey test. *: Abnormally distributed data expressed as median (25–75 percentile) and significance between groups was calculated using Kruskal-Wallis H test followed by Mann–Whitney U test. ^a^*p*: significance versus control; ^b^*p*: significance versus T2DM, ^c^*p*: significance versus T2DM+SAHA.

### Glycemic control and lipid profile

Fasting blood glucose, serum insulin and HOMA-IR levels were significantly elevated in T2DM group compared with the control group. Treatment with SAHA significantly reduced serum insulin levels and HOMA-IR compared with both the T2DM group and the Dapa-treated group. Meanwhile, Dapa significantly reduced HOMA-IR compared with the T2DM group and markedly decreased blood glucose levels compared with both the T2DM and SAHA-treated groups (Table 2). Dapa showed a more pronounced effect on hyperglycemia than SAHA; however, SAHA showed a more pronounced effect at normalizing insulinemia and addressing the underlying IR in this T2DM model. Serum levels of TGs, TC, LDL-C, and atherogenic index were significantly increased, while HDL-C levels were markedly decreased in T2DM group. However, both SAHA and Dapa significantly ameliorated all these alterations, with insignificant changes between SAHA and Dapa groups except for TC levels which were significantly decreased in the SAHA-treated compared with Dapa-treated groups (Table 3).

**Table 2:** Glycemic control in different studied groups.

Groups Parameters	Control	T2DM	T2DM+SAHA	T2DM+Dapa
*Blood glucose mg/dl (3rd week)	76.50 (71.50–81.00)	413.50 (360.25–420.25.25.25)	410.00 (364.00 −456.25)	425.00 (360.00–466.00)
Significance	*-*	^*a*^*p*=0.001	^*a*^*p*=0.001 ^*b*^*p*=0.42	^*a*^*p*=0.001 ^*b*^*p*=0.39 ^*c*^*p*=0.78
*Blood glucose mg/dl (11th week)	80.00 (69.50- 85.75)	343.00 (333.75–362.00)	334.00 (328.00 −365.00)	265.00 (250.00–284.00)
Significance		^*a*^*p*=0.001	^*a*^*p*=0.001 ^*b*^*p*=0.42	^*a*^*p*=0.001 ^*b*^*p*=0.003 ^*c*^*p*=0.003
Insulin (µIU/ml)	21.03 ± 0.99	34.33 ± 1.19	23.48 ± 2.01	35.03 ± 2.75
Significance		^*a*^*p*<0.0001	^*a*^*p*=0.066 ^*b*^*p*<0.0001	^a^*p*<0.0001, ^b^*p*=0.898^c^*p*<0.0001
HOMA-IR	3.98 ± 0.27	29.33 ± 1.39	19.29 ± 1.30	23.08 ± 1.69
Significance		^*a*^*p*<0.0001	^*a*^*p*<0.0001 ^*b*^*p*<0.0001	^*a*^*p*<0.0001 ^*b*^*p*<0.0001 ^*c*^*p*<0.0001

Normally distributed Data expressed as mean ± standard deviation and significance between groups was calculated using One-Way ANOVA followed by Tukey test. *: Abnormally distributed data expressed as median (25–75 percentile) and significance between groups was calculated using Kruskal-Wallis H test followed by Mann–Whitney U test. HOMA-IR: homeostatic model assessment of insulin resistance. ^a^*p*: significance versus control; ^b^*p*: significance versus T2DM, ^c^*p*: significance versus T2DM+SAHA.

**Table 3:** Lipid profile in different studied groups.

Groups Parameters	Control	T2DM	T2DM+SAHA	T2DM+Dapa
TGs (mg/dl)	88.00 ± 11.32	281.75 ± 34.0	179.72 ± 11.63	188.45 ± 9.86
Significance	-	^*a*^*p*<0.0001	^a^*p*<0.0001, ^b^*p*<0.0001	^a^*p*<0.0001, ^b^*p*<0.0001, ^c^*p*=0.822
*TC (mg/dl)	123.12 (105.65 −131.69)	335.12 (303.17 −382.31)	136.43 (133.40 −139.56)	229.66 (210.18 −231.05)
Significance	-	^*a*^*p*=0.001	^*a*^*p*=0.001 ^*b*^*p*=0.004	^*a*^*p*=0.001 ^*b*^*p*=0.003 ^*c*^*p*=0.014
*LDL-C (mg/dl)	75.06 (63.14–80.02)	172.15 (163.45 −191.47)	136.43 (133.40 −139.56)	146.96 (129.38 −149.11)
Significance	-	^*a*^*p*=0.001	^*a*^*p*=0.001 ^*b*^*p*=0.004	^*a*^*p*=0.001 ^*b*^*p*=0.003 ^*c*^*p*=0.250
HDL-C (mg/dl)	55.91 ± 4.93	28.93 ± 4.06	44.21 ± 3.69	41.23 ± 2.31
Significance	-	^*a*^*p*<0.0001	^*a*^*p*<0.0001, ^*b*^*p*<0.0001	^a^*p*<0.0001, ^b^*p*<0.0001, ^c^*p*=0.550
Atherogenic index	0.19 ± 0.09	0.99 ± 0.12	0.61 ± 0.06	0.66 ± 0.04
Significance	-	^*a*^*p*<0.0001	^a^*p*<0.0001, ^b^*p*<0.0001	^a^*p*<0.0001, ^b^*p*<0.0001 ^*c*^*p*=0.674

Normally distributed data expressed as mean ± standard deviation and significance between groups was calculated using One-Way ANOVA followed by Tukey test. *: Abnormally distributed data expressed as median (25–75 percentile) and significance between groups was calculated using Kruskal-Wallis H test followed by Mann–Whitney U test. TC: total cholesterol, TGs: triglycerides, LDL-C: low-density lipoprotein cholesterol, HDL-C: high-density lipoprotein cholesterol.  ^a^*p*: significance versus control; ^b^*p*: significance versus T2DM, ^c^*p*: significance versus T2DM+SAHA.

### Cardiac enzymes and oxidative stress markers

Levels of CK-MB, LDH, and MDA were significantly increased, while SOD levels were significantly reduced in the T2DM group, indicating a marked oxidative stress state that likely contributes to myocardial injury. Treatment with either SAHA or Dapa significantly improved all these parameters with insignificant changes between SAHA-treated and Dapa-treated groups (Table 4). These findings suggest that both agents exert comparable effects in attenuating OS and myocardial injury under the studied conditions.

**Table 4:** Cardiac enzymes and oxidative stress markers in different studied groups.

Groups Parameters	Control	T2DM	T2DM+SAHA	T2DM+Dapa
SOD (U/gm)	3.61 ± 0.32	0.89 ± 0.12	1.86 ± 0.20	1.66 ± 0.15
Significance	-	^*a*^*p*<0.0001	^a^*p*<0.0001, ^b^*p*<0.0001	^a^*p*<0.0001, ^b^*p*<0.0001,^c^*p*=0.424
MDA (nmol/gm)	1.03 ± 0.14	4.07 ± 0.30	1.81 ± 0.18	2.02 ± 0.26
Significance	-	^*a*^*p*<0.0001	^a^*p*<0.0001, ^b^*p*<0.0001	^a^*p*<0.0001, ^b^*p*<0.0001, ^*c*^*p*=0.333
*CK-MB (ng/mL)	0.32 (0.29–0.35)	2.37 (1.99–2.87)	1.06 (1.03–1.11)	1.12 (1.02–1.15)
Significance	-	^*a*^*p*=0.001	^*a*^*p*=0.001 ^*b*^*p*=0.004	^*a*^*p*=0.001 ^*b*^*p*=0.003 ^*c*^*p*=0.114
*LDH (U/L)	283.27 (264.76–302.75.76.75)	785.67 (761.02 −801.02)	597.06 (573.77–686.12.77.12)	611.65 (592.94 −687.08)
Significance	-	^*a*^*p*=0.001	^*a*^*p*=0.001 ^*b*^*p*=0.004	^*a*^*p*=0.001 ^*b*^*p*=0.003 ^*c*^*p*=0.114

Normally distributed data expressed as mean ± standard deviation and significance between groups was calculated using One-Way ANOVA followed by Tukey test. *: Abnormally distributed data expressed as median (25–75 percentile) and significance between groups was calculated using Kruskal-Wallis H test followed by Mann–Whitney U test. SOD: superoxide dismutase, MDA: malondialdehyde, CK-MB: creatine kinase-MB, LDH: lactate dehydrogenase. ^a^*p*: significance versus control; ^b^*p*: significance versus T2DM, ^c^*p*: significance versus T2DM+SAHA.

### Assessment of cardiac injury and remodeling

**Fig. 1:**
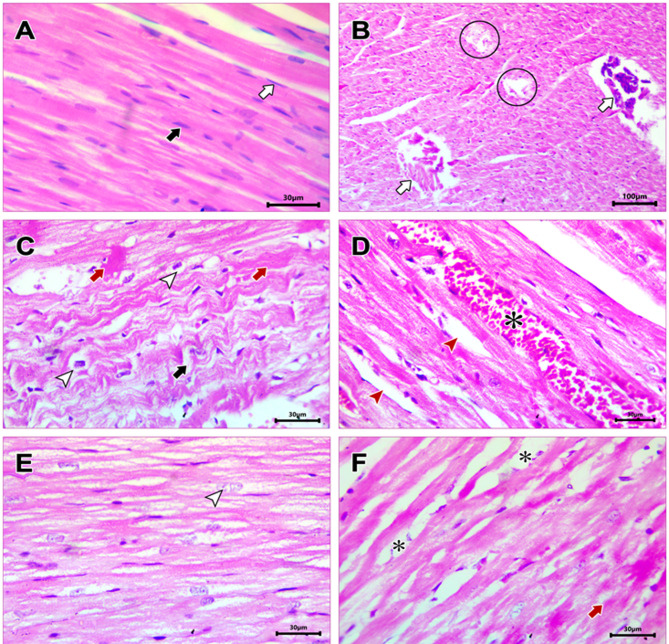
Photomicrographs of H&E-stained myocardial sections of different groups (**A**): Control group showing striated muscle fibers which appear regularly arranged with acidophilic cytoplasm, central oval nuclei (Black arrow) surrounded by narrow areas of endomysium containing fibroblast (White arrow) [x400 scale bar 30 μm]. (**B**): T2DM group showing disorganized cardiac muscle fibers with myofiber degeneration (White arrow) and characteristic moth-eaten appearance in some areas (Circle) [x100, Scale bar 100 μm]. (**C**): T2DM group showing wavy cardiac muscle fibers (Black arrow) with sarcoplasmic vacuolation (White arrowhead) and homogeneous, deeply acidophilic areas devoid of nuclei (Red arrow) [x400, Scale bar 30 μm]. (**D**): T2DM group revealing wide inter-fiber spaces (Red arrowhead), and vascular congestion (Asterisk) [x400, Scale bar 30 μm]. (**E**) T2DM+SAHA group displaying apparently normal histological features of cardiac muscle. Cardiomyocytes have acidophilic sarcoplasm with transverse striations and centrally located pale nuclei (White arrowhead) [H & E x400, Scale bar 30 μm]. (**F**) T2DM+Dapa group showing partial restoration of normal architecture. Deeply acidophilic fibers devoid of nuclei (Red arrow), and obvious edematous changes are also observed (Asterisk) [x 400, Scale bar 30 μm].

Microscopic examination of cardiac muscle in T2DM group showed marked disruption of the normal architecture of the myocardium with focal areas of degeneration (Fig. 1.B). Additionally, congested myocardial blood vessels and widened inter-fiber spaces were observed (Fig. 1.D). SAHA treatment restored normal histological architecture to the cardiac muscle except for some areas of focal degeneration and wide inter-fiber spaces (Fig. 1.E). Dapa treatment regressed some of these T2DM-induced histological alterations; however sarcoplasmic vacuolation, wide intercellular spaces, and focal areas of degeneration were also observed (Fig. 1.F). The reduction in histological lesions and atrophy suggests a preservation of cellular integrity against glucose-induced cytotoxicity. While Dapa provides protection likely through systemic glycemic management, SAHA’s more pronounced effect may be attributed to a direct epigenetic modulation of gene expression and protein regulatory pathways.

**Fig. 2:**
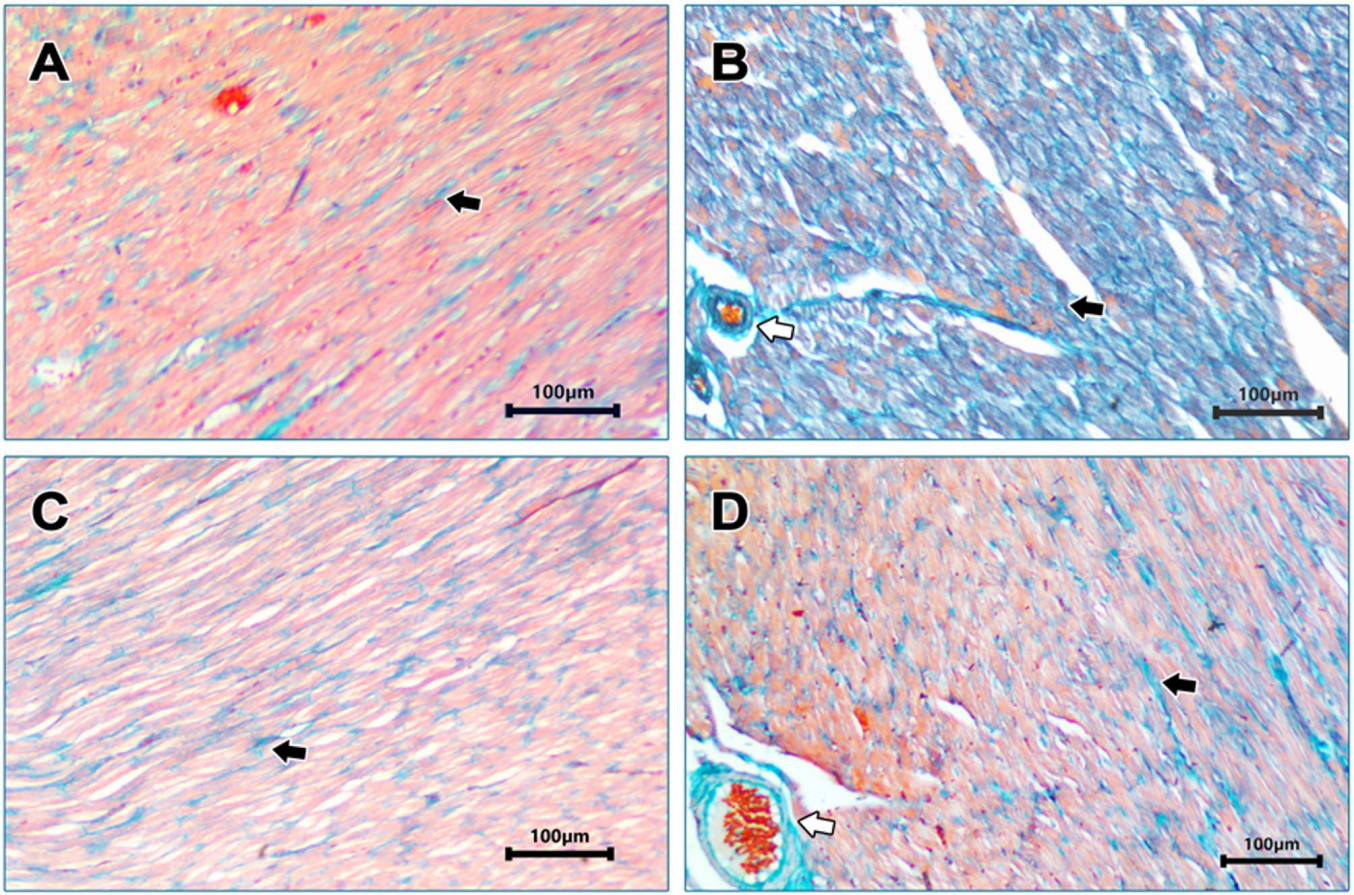
Photomicrographs of Masson’s Trichrome-stained myocardial tissues of different groups: (**A**): Control group showing fine blue stained collagen fibers (Black arrow). (**B**): Diabetic group showing extensive collagen fibers deposition (Black arrow), and perivascular fibrosis (White arrow). (**C**): T2DM+SAHA revealing mild collagen fibers deposition (Black arrow). (**D**): T2DM+Dapa group revealing moderate collagen fibers deposition (Black arrow), and perivascular fibrosis (White arrow). [x 100, Scale bar 100 μm].

In Masson’s Trichrome-stained sections, examination of the T2DM group revealed extensive collagen fibers deposition with perivascular fibrosis (Fig. 2.B). Meanwhile examination of SAHA-treated group showed mild collagen fibers deposition (Fig. 2.C), and examination of Dapa-treated group showed moderate collagen fibers deposition and perivascular fibrosis (Fig. 2.D).

**Fig. 3:**
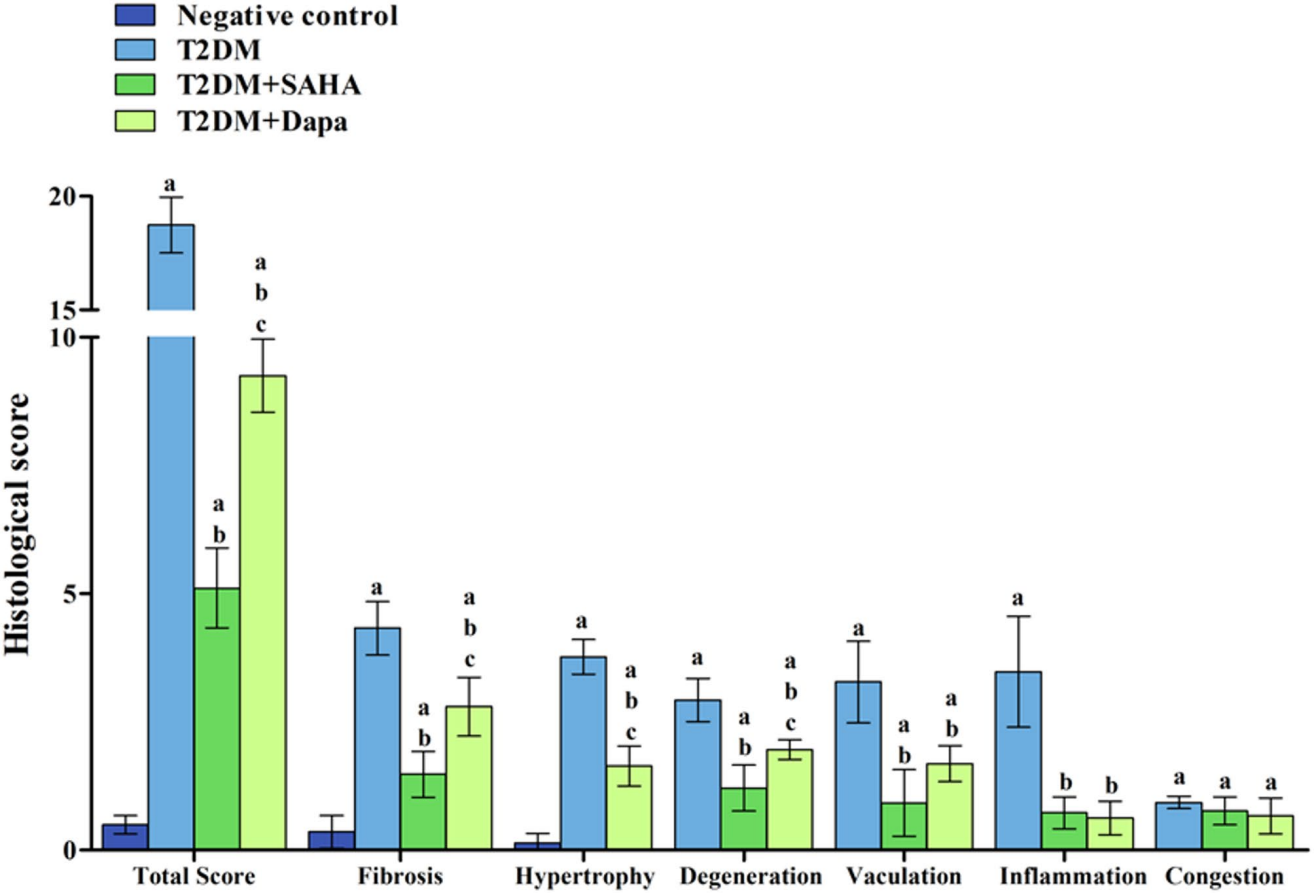
Semi-quantitative histological lesion scoring in cardiac tissues of different groups. Data expressed as mean ± standard deviation. Significance between groups was calculated using One-Way ANOVA followed by Tukey test a: significance versus control. b: significance versus T2DM. c: significance versus T2DM+SAHA. Control group scored zero on the following parameters: degeneration, vacuolation, inflammation and congestion. While T2DM+SAHA group scored zero on hypertrophy.

Morphometric analysis revealed that percentage area of collagen fibrosis, cardiomyocyte cross-sectional diameter (µm), and semiquantitative histopathological scoring of cardiac lesions were elevated in T2DM group (Fig. 3). Administration of either SAHA or Dapa considerably decreased cardiac fibrosis, hypertrophy, and injury. Moreover, SAHA treatment substantially outperformed Dapa and significantly decreased all these parameters, in addition to returning cell diameters to normal levels (Table 5). The significant reduction in interstitial fibrosis implies that both agents interfere with the activation of cardiac fibroblasts, yet the greater effect observed with SAHA may indicate a more direct role in limiting fibroblast-to-myofibroblast differentiation.

**Table 5:** Morphometric analysis of heart and Cx43 protein and mRNA expression in ventricular tissues in different studied groups.

Groups Parameters	Control	T2DM	T2DM+SAHA	T2DM+Dapa
Scoring of lesions	0.49 ± 0.18	18.46 ± 1.30	5.11 ± 0.78	9.67 ± 0.39
Significance	-	^a^*p*<0.0001	^a^*p*<0.0001, ^b^*p*<0.0001	^a^*p*<0.0001, ^b^*p*<0.0001, ^c^*p*<0.0001
Cardiomyocyte diameter (µm)	18.21 ± 0.09	26.92 ± 0.57	18.78 ± 0.23	23.45 ± 0.39
Significance	-	^a^*p*<0.0001	^a^*p*=0.068, ^b^*p*<0.0001	^a^*p*<0.0001, ^b^*p*<0.0001, ^c^*p*<0.0001
% Area of collagen fibrosis	8.34 ± 0.87	51.32 ± 2.25	17.42 ± 1.13	34.92 ± 2.54
Significance	-	^a^*p*<0.0001	^a^*p*<0.0001 ^b^*p*<0.0001	^a^*p*<0.0001, ^b^*p*<0.0001, ^c^*p*<0.0001
% Area of Cx43 protein expression	1.97 ± 0.14	0.53 ± 0.07	4.80 ± 0.22	2.54 ± 0.16
Significance	-	^a^*p*<0.0001	^a^*p*<0.0001, ^b^*p*<0.0001	^a^*p*<0.0001, ^b^*p*<0.0001, ^c^*p*<0.0001
Cx43 mRNA fold change	1.02 ± 0.06	0.27 ± 0.04	8.00 ± 0.17	2.45 ± 0.17
Significance	-	^a^*p*<0.0001	^a^*p*<0.0001, ^b^*p*<0.0001	^a^*p*<0.0001, ^b^*p*<0.0001, ^c^*p*<0.0001

Normally distributed data expressed as mean ± standard deviation and significance between groups was calculated using One-Way ANOVA followed by Tukey test. Cx43: connexin-43. ^a^*p*: significance versus control; ^b^*p*: significance versus T2DM, ^c^*p*: significance versus T2DM+SAHA.

### Cx43 expression in ventricular tissue

**Fig. 4:**
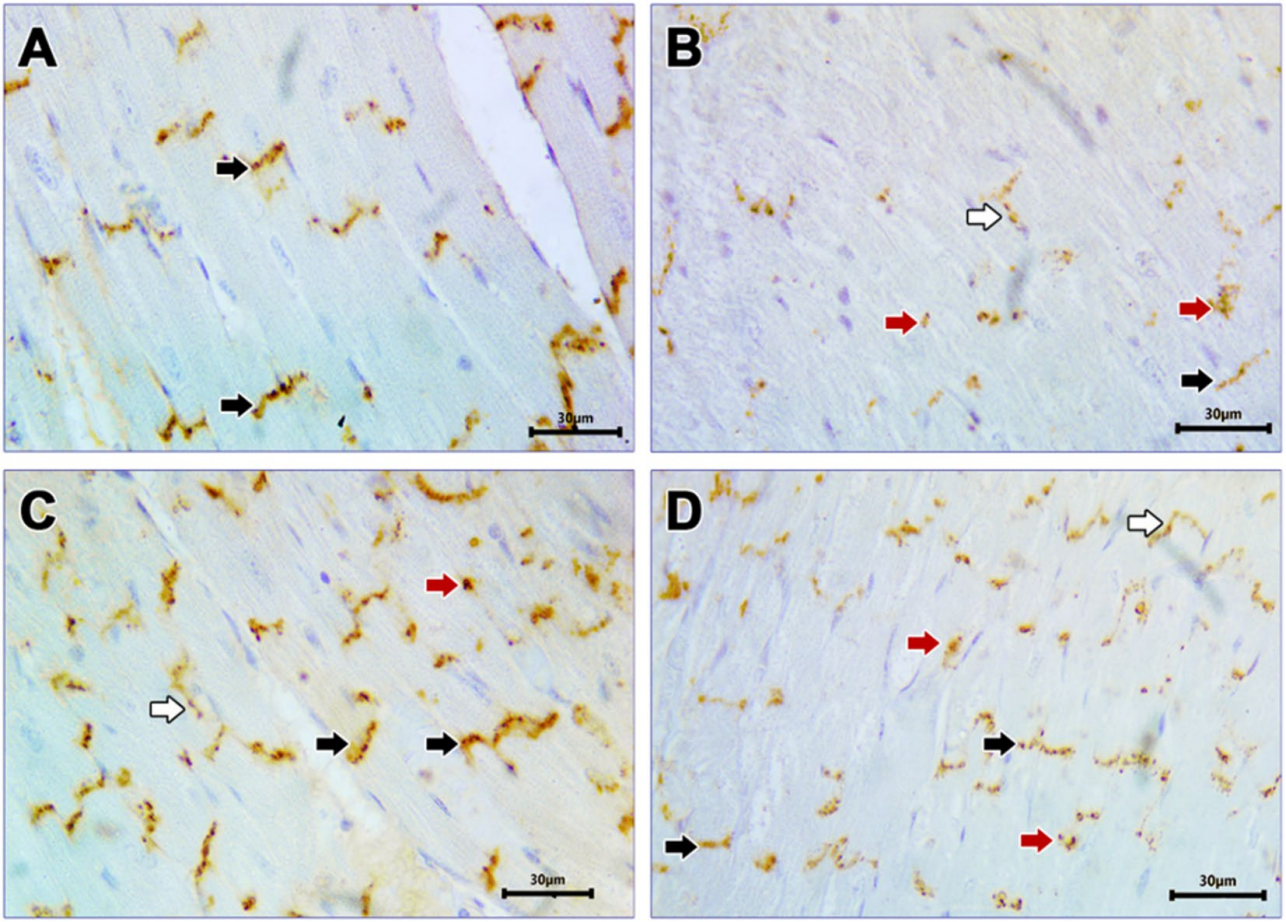
Cx43 immune-stained myocardial sections of different groups. Photomicrographs of Anti-Cx43 immune-stained longitudinal myocardial tissues. (**A**): Control group revealing Cx43 strongly positive immune reaction with apparent normal distribution at the IDs (black arrow) (**B**): T2DM group revealing weak positive Cx43 immune reaction, with apparent lateralization away from the IDs (white arrow), and abnormal distribution (Red arrow). Some minimal areas appear to retain normal distribution at the IDs (black arrow). **C**: T2DM+SAHA diabetic group revealing strong positive Cx43 immune reaction, with apparent normal distribution at the IDs (Black arrow), except for some areas which show lateralization (White arrow) and abnormal distribution (Red arrow). **D**: T2DM+Dapa group showing strong positive Cx43 immune reaction, with lateralization (White arrow) and abnormal distribution (Red arrow). Areas of apparent normal distribution at the IDs are also observed (black arrow) [x 400. Scale bar 30 μm].

Examination of anti-Cx43 immune-stained sections of controls revealed strong immune positive reaction, with normal distribution localized to the IDs (Fig. 4.A). Examination of the T2DM group showed weak immune positive reaction, with abnormal distribution and lateralization to lateral membrane of cardiomyocytes (Fig. 4.B). Examination of SAHA-treated group revealed strong immune positive reaction with apparent normal distribution at the IDs, except for some areas of abnormal distribution (Fig. 4.C). The increased localization of Cx43 at the IDs following SAHA treatment indicates a restoration of proper gap junction assembly, which is essential for maintaining synchronized electrical conduction in the myocardium. Examination of the Dapa-treated group revealed strong immune positive reaction with apparent lateralization and internalization, while some areas demonstrated normal distribution at the IDs (Fig. 4.D). The area percentage of Cx43 immunoreactivity and Cx43 mRNA expression were significantly reduced in the T2DM group. Meanwhile, administration of either SAHA or Dapa significantly increased Cx43 expression compared with both control and T2DM groups. Furthermore, SAHA considerably increased Cx43 levels compared with Dapa (Table 5). Notably, treatment with SAHA shifted Cx43 expression toward baseline levels, reflecting a near-normalization of the cardiac protein profile and highlighting the therapeutic potential of this intervention in reversing T2DM-induced damage.

### Discussion

Diabetic cardiomyopathy represents a major and growing health burden, with reported incidence rates reaching up to 23% in 2024 and continuing to rise^22^. Despite its clinical significance, the precise molecular mechanisms underlying DCM remain largely ambiguous, and consequently, no definitive preventive or therapeutic strategies specifically targeting DCM are currently available.

In the present study, all T2DM groups displayed marked dyslipidemia and impaired glycemic control, in the form of significantly reduced serum HDL-C levels alongside elevated serum glucose, insulin, HOMA-IR, TC, TGs, LDL-C, and atherogenic indices. These results are supported by previous literature on T2DM (STZ/HFD) models^23^. Hyperglycemia following STZ administration is attributed to its cytotoxic effects on pancreatic β-cells, while IR develops secondary to elevated circulating free fatty acids induced by HFD consumption^24^. This subsequently disrupts hepatic functions and affects key lipases involved in the regulation of lipoprotein levels, ultimately leading to dyslipidemia^25^. The observed metabolic disturbances and oxidative stress in the diabetic group are consistent with established mechanisms underlying DCM, where hyperglycemia and IR promote oxidative damage, inflammation, and myocardial remodeling^23–27^.

Both SAHA and Dapa in this research significantly improved glycemic control and lipid profiles compared with the T2DM group. Notably, SAHA significantly outperformed Dapa in ameliorating TC levels, hyperinsulinemia, and IR, whereas Dapa caused a more pronounced reduction in serum glucose levels. These findings align with previous studies in T2DM models reporting that HDACIs improve glycemic control and lipid metabolism^28^. SAHA is associated with pathways involved in insulin sensitivity through upregulation of key components of the insulin signaling pathway, such as glucose transporter-4 and insulin receptor substrate-1^29^, as well as modulation of AMP-activated protein kinase signaling^30^. Additionally, SAHA was shown to ameliorate dyslipidemia by suppressing genes involved in cholesterol uptake and synthesis^31^. The beneficial effects of Dapa on glycemic control and lipid metabolism in DCM are well documented^32^. As a potent glucosuric agent, Dapa lowers blood glucose levels; additionally, it improves IR and dyslipidemia through weight reduction, enhanced lipid oxidation, and attenuation of OS^25^. Both SAHA and dapagliflozin improved metabolic and oxidative parameters, consistent with previous studies demonstrating their cardioprotective roles in diabetic models^28,32^. SAHA has been shown to enhance insulin signaling and lipid metabolism through modulation of key metabolic pathways^29–31^, while dapagliflozin exerts its effects through glycosuria, improved lipid utilization, and reduction of oxidative stress^25,32^.

In the current study, histopathological examination of cardiac tissues in the T2DM group revealed severe disruption of myocardial architecture, accompanied by significant cardiomyocyte hypertrophy and fibrosis. These structural abnormalities were associated with elevated cardiac homogenate MDA levels, increased serum CK-MB and LDH, and a significant reduction in cardiac homogenate SOD activity. These observations are consistent with earlier studies of T2DM models, which reported comparable histological alterations, elevated cardiac enzymes^23,24^, and increased OS levels^26^. Hyperglycemia, IR, and OS are typically considered the major culprits in DCM development, inducing cardiomyocyte injury through activation of pro-apoptotic pathways and directly damaging cellular proteins and DNA^27^. Moreover, studies indicate that these factors promote myocardial hypertrophy and fibrosis by dysregulating matrix metalloproteinases, activating hypertrophy-associated HDACs^33^, and upregulating profibrotic growth factors such as transforming growth factor-β1^24^.

Treatment with either SAHA or Dapa markedly attenuated cardiac remodeling and significantly reduced cardiac enzymes and OS markers. Remarkably, SAHA completely reversed cardiomyocyte hypertrophy, and significantly reduced fibrosis and cardiac injury compared with Dapa. To the best of our knowledge, this is the first study to demonstrate the effects of SAHA on CK-MB and LDH levels in a DCM model. Previous studies in T2DM models have reported that HDACIs reduce OS and alleviate adverse cardiac remodeling^28^. These studies attributed the antioxidant effects of SAHA to the upregulation of antioxidant genes (e.g., SOD2), and activation of nuclear factor erythroid 2–related factor 2 (NRF2), which is generally considered the principal regulator of antioxidant defense^34^. Furthermore, SAHA potentially mitigates cardiac injury by stimulating angiogenesis and inhibiting apoptosis^30^, while its potent antihypertrophic effects are largely mediated through inhibition of hypertrophy-promoting HDACs^33^. Similarly, Dapa has been shown to significantly reduce OS^13^, improve cardiac remodeling, and decrease CK-MB and LDH levels in DCM models^32^, possibly through inhibition of apoptosis, suppression of transforming growth factor-β expression, and downregulation of collagen I and III gene expression^33^. Previous literature also has linked Dapa with pathways involved in enhancing autophagy and upregulating NRF2 expression^13^. Oxidative stress plays a central role in myocardial injury by damaging cellular proteins, lipids, and DNA, ultimately contributing to cardiac dysfunction^26,27^. The reduction in MDA and increase in SOD observed in treated groups reflect attenuation of oxidative stress.

The present study further demonstrated a marked reduction in Cx43 mRNA and protein expression in the T2DM group, accompanied by lateralization of Cx43 away from IDs. These findings are consistent with previous reports describing reduced Cx43 protein expression with abnormal distribution^35^ and decreased Cx43 mRNA expression in the myocardium of T2DM rats^36^. T2DM disrupts Cx43 transcription directly via histone deacetylation and indirectly through hyperglycemia, IR, and OS. Additionally, T2DM impairs the trafficking of Cx to the IDs, contributing to Cx43 lateralization and enhanced proteolysis^37^. In contrast, some studies have reported unaltered^38^ or even elevated^39^ Cx43 mRNA levels in DCM models, with these discrepancies potentially attributed to differences in experimental design, including the use of type 1 diabetes models or lack of ventricular-specific analysis.

In the present research, both SAHA and Dapa significantly increased Cx43 mRNA and protein expression and restored its proper localization at the IDs. Notably, SAHA significantly surpassed Dapa in all these parameters. To the best of our knowledge, this is the first study to investigate the effects of SAHA or Dapa on Cx43 mRNA expression in the myocardium of T2DM rats. As a potent HDACI, SAHA directly enhances Cx43 transcription through chromatin hyperacetylation and indirectly improves Cx43 expression and localization by reducing OS, inflammation, and IR^40^. Meanwhile, some studies have reported that HDACIs have no effect on Cx43 protein expression in cardiac hypertrophic mice^41^ and cultured ventricular myocytes^42^. Whereas, others have demonstrated reduced Cx43 mRNA but increased protein expression in cultured smooth muscle cells^5^. These conflicting results likely reflect differences in underlying pathophysiology, cellular context, or activation of compensatory feedback mechanisms. Meanwhile, SGLT2is have been shown to improve Cx43 protein expression and localization in DCM models^43^, and to enhance Cx43 mRNA expression in sepsis-induced cardiac injury^43^. The upregulation of Cx43 mRNA by Dapa is likely to be indirect; occurring through its ameliorative effect on hyperglycemia and its sequelae of inflammation and OS, which ultimately improve the process of transcription, upregulate Cx43 protein expression, and restore proper Cx43 localization^6,7^.

The observed restoration of Cx43 expression and localization in the treated groups can be mechanistically explained by both epigenetic and redox-dependent pathways. As a histone deacetylase inhibitor, SAHA promotes chromatin hyperacetylation, leading to transcriptional activation of the GJA1 gene encoding Cx43. This direct epigenetic regulation likely accounts for the marked increase in Cx43 mRNA expression observed in the present study^4,40^. In addition, the significant reduction in oxidative stress, evidenced by decreased malondialdehyde and increased superoxide dismutase levels, may contribute to improved Cx43 protein stability and proper trafficking to intercalated discs, thereby preserving gap junction integrity^26,27,37^.

In contrast, dapagliflozin appears to exert its effects on Cx43 predominantly through indirect mechanisms, including improvement of hyperglycemia, insulin resistance, and oxidative stress^6,25^. While these effects create a more favorable cellular environment for Cx43 expression and function, they do not directly target transcriptional regulation. This distinction may explain the comparatively greater efficacy of SAHA in restoring both Cx43 expression and its physiological localization. Collectively, these findings suggest that combined modulation of epigenetic regulation and oxidative stress plays a critical role in preserving gap junction integrity in diabetic cardiomyopathy. Importantly, the restoration of Cx43 localization at intercalated discs suggests potential improvement in electrical coupling between cardiomyocytes, as proper Cx43 distribution is essential for coordinated impulse propagation^44^. Conversely, the lateralization of Cx43 observed in the diabetic group is indicative of gap junction dysfunction, which may contribute to impaired electrical conduction and increased arrhythmogenic risk in diabetic cardiomyopathy^35,36,44^ The superior effect of SAHA compared to dapagliflozin may be attributed to its dual mode of action, involving both direct epigenetic regulation of gene expression and indirect antioxidative effects. While dapagliflozin primarily improves metabolic parameters, SAHA directly targets transcriptional pathways, which may explain the more pronounced restoration of Cx43 expression and localization.

The reduction and lateralization of Cx43 observed in the diabetic group are indicative of impaired gap junction communication, which is known to disrupt electrical coupling between cardiomyocytes. Such alterations may contribute to conduction abnormalities and increased arrhythmogenic risk in diabetic cardiomyopathy. The restoration of Cx43 localization at intercalated discs following treatment suggests potential improvement in electrical coupling, although functional confirmation through electrophysiological assessment was not performed. Conflicting findings regarding Cx43 expression in diabetic models have been reported, likely due to differences in experimental design, disease models, and tissue specificity^38,39^.

## Conclusions

The present study provides novel insights into the potential involvement of epigenetic mechanisms in the pathogenesis of DCM. The findings suggest that T2DM may be associated with altered ventricular Cx43 mRNA expression in the STZ/HFD rat model. Both SAHA and dapagliflozin demonstrated protective effects against diabetic cardiomyopathy in this experimental model. SAHA showed comparatively greater effects on Cx43 expression and cardiac remodeling; however, these findings should be interpreted cautiously given the study limitations. Further studies are required to validate these results and explore underlying mechanisms and potential clinical relevance.

### Limitations of the study

The present study has several limitations that warrant consideration. First, the lack of functional cardiac assessments, such as echocardiography and electrocardiography, precludes a definitive correlation between the observed molecular improvements in Cx43 and actual contractile or rhythmic performance. While histological and molecular data strongly suggest structural recovery, the absence of functional parameters remains a significant constraint in fully characterizing the degree of reversal in DCM-associated dysfunction. Unfortunately these techniques were not available at our institution Second, this study utilized a single-dose regimen for both SAHA and Dapa. While the doses were selected based on efficacy demonstrated in previous literature, the lack of a multi-dose escalation precludes the determination of a clear dose-response relationship. Consequently, the therapeutic window and optimal dosing for maximizing Cx43 restoration remain to be elucidated. Finally, the study design did not include non-diabetic groups treated with SAHA or Dapa. The absence of these baseline treatment controls limits our ability to distinguish between the drugs’ specific anti-diabetic effects and their potential baseline physiological impacts on healthy myocardial tissue. Future research incorporating functional diagnostics, dose-dependency trials, and non-diabetic treated cohorts is essential to validate these findings and clarify the clinical potential of these interventions.

## Data Availability

All data available with the corresponding author on request.
